# Anorectal Gastrointestinal Stromal Tumor: A Case Report and Literature Review

**DOI:** 10.1155/2013/934875

**Published:** 2013-03-25

**Authors:** Sanjeev Singhal, Anu Singhal, Rahul Tugnait, Vineet Varghese, Bishwanath Tiwari, Pankaj K. Arora, Pawan Malik, Mriganka Deuri Bharali, Ankur Subhash Dhuria, Pushkar Chauhan, Chandrakant Singh, Amit Ballani, Vishnu Panwar

**Affiliations:** ^1^Department of Surgery, Northern Railway Central Hospital, New Delhi, India; ^2^Department of Radiology, ESI Model Hospital and PGIMSR, Basaidarapur, New Delhi 110001, India; ^3^Department of Radiology, Northern Railway Central Hospital, New Delhi, India; ^4^Department of Anaesthesia, Northern Railway Central Hospital, New Delhi, India

## Abstract

Gastrointestinal stromal tumors or “GIST” are mesenchymal neoplasms expressing KIT(CD117) tyrosine kinase and showing the presence of activating mutations in KIT or *PDGFR**α*** (platelet-derived growth factor alpha). GIST of anal canal is an extremely rare tumor, accounting for only 3% of all anorectal mesenchymal tumors and 0.1–0.4% of all GIST. GIST with large tumor size and high mitotic activity are highly malignant, but the biological behavior of anorectal GIST is less clear. Abdominoperineal resection (APR) or conservative surgery is the best treatment option. Imatinib mesylate, a tyrosine kinase inhibitor, has shown promising results in its management. We present a case of anorectal GIST diagnosed by computed tomography (CT) scan, magnetic resonance imaging (MRI), and colonoscopy with biopsy. The patient underwent abdominoperineal resection (APR) and was confirmed on histopathology to have anal canal GIST with tumor size more than 5 cm in maximum dimension and mitotic figures more than 5/50 high power field (HPF). The CD117—immunoreactive score—was 3+ in spindled cells. Therefore the patient was put on adjuvant imatinib mesylate 400 mg daily.

## 1. Introduction

Gastrointestinal stromal tumor or “GIST” was a name given in 1983 to a group of gastrointestinal tumors which were otherwise unclassifiable as being of smooth muscle or neurogenic origin [1]. They are mesenchymal neoplasms expressing KIT(CD117) tyrosine kinase and showing presence of activating mutations in KIT or *PDGFRα* (platelet-derived growth factor alpha) [2]. It is the commonest gastrointestinal mesenchymal tumor [3] with the commonest site being stomach (50–60%), followed by small intestine (30–40%), colon (7%), and oesophagus (1%) [4]. GIST of anal canal and rectum are often grouped together and account for nearly 5% of all GIST [4, 5]. However, of these only 2–8% are from anal canal, making GIST of anal canal an extremely rare tumor [6, 7].

## 2. Case Report

A 61-year-old male presented with pain during defecation and occasional bleeding per rectum over 2 months. Pain was nonradiating, dull aching, and persistent. Pain increased with constipation. Bleeding was frank red and came as drops after passage of stool. There was no tenesmus. There was no dizziness, weakness, pica, or weight loss. There was no significant relief with medication.

The patient was not a known case of piles/diabetes mellitus/hypertension/tuberculosis/or any other chronic ailment. The patient was a known alcoholic and smoker. He had no urinary complaints. There were no other complaints referable to chest and cardiac or nervous system.

He was a heterosexual with no known drug allergies.

On examination he was moderately built and of average nourishment. Karnofsky performance scale was more than 80. There was no pallor/icterus/pedal edema/lymphadenopathy. On digital rectal examination, a hard indurated nontender mass was felt from 2 o'clock to 9 o'clock, starting from the anal verge. The mass had a bosselated surface, and the overlying mucosa was tethered. The upper border of the mass could not be reached. No inguinal, iliac, para aortic, or supraclavicular lymph nodes were palpable.

**Figure 1:**
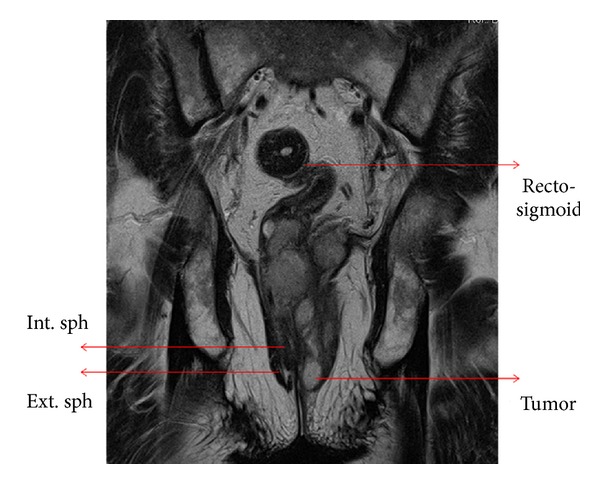
T2W sagittal section showing tumor extending up to anal verge and involving left wall of anal canal with loss of sphincteric anatomy.

**Figure 2:**
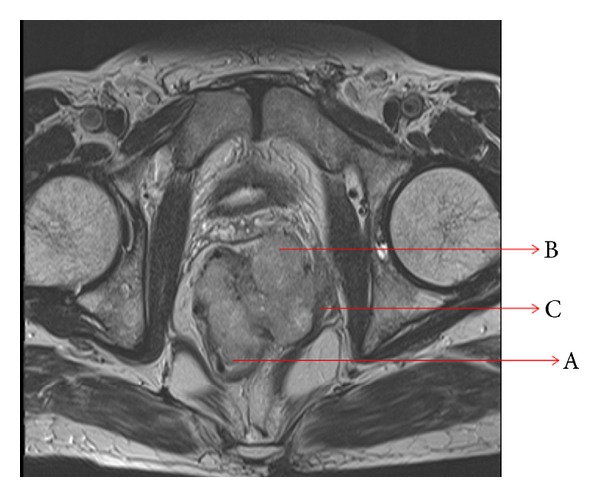
T2W axial image through distal rectum showing partly endophytic (A) and partly exophytic (B) mass having intermediate signal intensity. Left puborectalis muscle is invaded, thickened, and retracted (C).

His hematological and biochemical parameters were all within normal limits, and he was HIV seronegative. His transrectal ultrasound revealed an enlarged prostate with insignificant postvoid residual urine. There was significant ill-defined mural thickness and hypervascularity along anorectal canal causing indentation of prostate. CT scan of the abdomen and pelvis showed an endo-exophytic soft tissue mass at anorectum suggestive of mitotic pathology. MRI pelvis revealed a lesion involving rectum and anal canal with extension into intersphincteric plane and puborectalis (Figures 1 and 2). His colonoscopy was suggestive of anorectal carcinoma, and colonoscopy guided biopsy was suggestive of spindle cell carcinoma. A repeat punch biopsy was suggestive of leiomyosarcoma.

**Figure 3:**
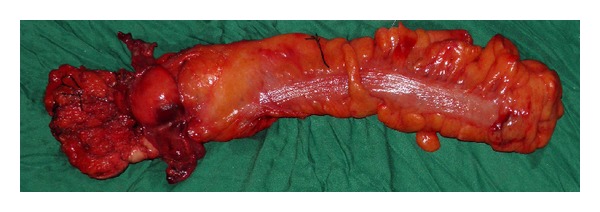
Abdominoperineal resection specimen.

**Figure 4:**
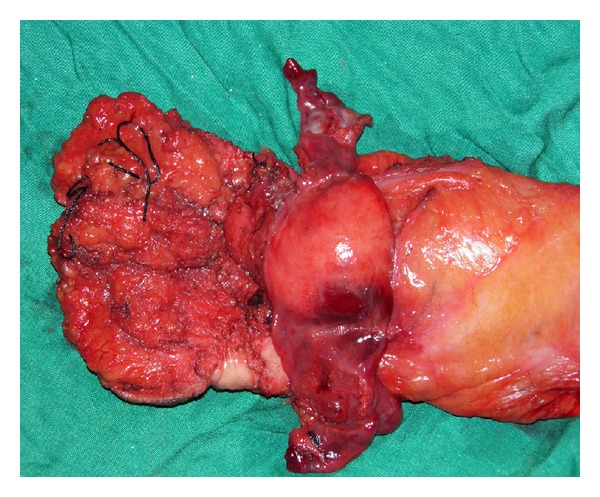
Growth in anorectal region.

**Figure 5:**
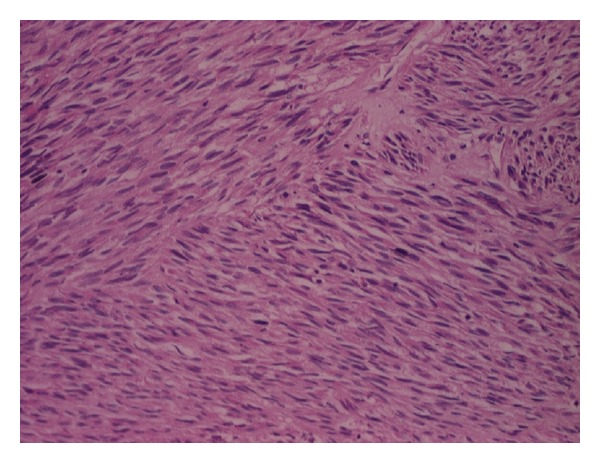
Spindle cells arranged in fascicles and showing mild nuclear pleomorphism and mitotic activity (100x magnification).

**Figure 6:**
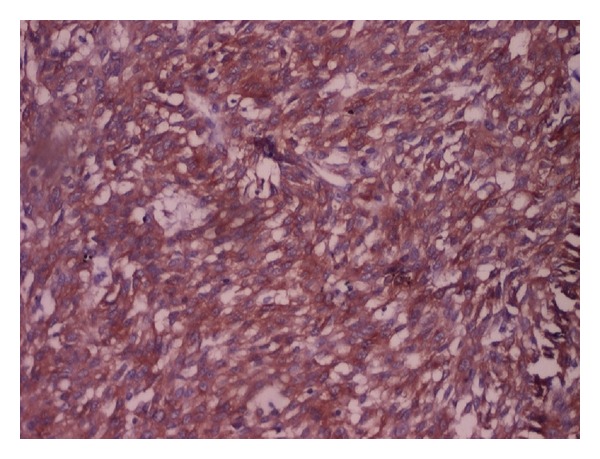
Spindle cells showing strong immunoreactivity for CD117 on IHC (score 3+ to 4+) (100x magnification).

The patient underwent abdominoperineal resection with end colostomy (Figures 3 and 4). Histopathological examination report revealed anal canal GIST with tumor size more than 5 cm in maximum dimension. Mitotic figures were more than 5/50 HPF. Proximal and distal resected margins were uninvolved by tumor. Circumferential resected margin was less than 0.1 cm away from tumor. There was no lymphovascular invasion. Out of the 6 lymph nodes resected none were involved by tumor. The CD117—immunoreactive score—was 3+ in spindled cells (Figures 5 and 6).

## 3. Discussion

The incidence of anal cancer in the western world is between 7 and 9 per million population. It contributes to only 1.5% of all malignancies of the digestive system [8]. Of these, GIST form only 3% of anorectal mesenchymal tumors [7]. However, the exact data in eastern world may be different, as in China, unlike in western countries, rectal cancer accounts for approximately 70% of colorectal cancers [9].

The anal canal extends from the perianal skin (anal verge) to the rectal mucosa. An important landmark within the canal is the dentate or pectinate line, which represents the end of the squamous mucosa and the beginning of a zone of transition from squamous to nonsquamous (either transitional or rectal glandular) mucosa. Thus, tumors arising in the anal canal can be either keratinizing or nonkeratinizing depending on their location in relation to the dentate line. Importantly, both keratinizing and nonkeratinizing tumors appear to have similar biology and prognosis [10]. Adenocarcinomas, on the other hand, behaves quite differently and should be treated like rectal cancers. Since there is no easily identifiable landmark between the rectum and anal canal, one has to rely on the pathologic classification of tumors in this area rather than the surgical or endoscopic classification [6].

GIST are currently thought to originate from interstitial cells of Cajal. The presence of interstitial Cajal-like cells has been reported in several extraintestinal organs including urinary bladder, prostate gallbladder, omentum, uterus, fallopian tube, and atrial and ventricular myocardium [11, 12]. This may explain the development of extraintestinal GIST [13, 14]. Mutational statuses of c-KIT and PDGFR*α* genes are the basis for the diagnosis of this neoplasia and represent the criteria for surgical therapy, expected chemotherapy response, and clinical outcomes [6].

Risk factors for anorectal malignancies include female sex, patients with history of human papilloma virus (HPV) infection, human immunodeficiency virus- (HIV-) positive patients, patients who engage in anal receptive intercourse, presence of sexually transmitted disease, a history of more than 10 sexual partners, and a history of cervical, vulval, or vaginal cancers. Immunosuppressed individuals including renal transplant patients and those on chronic glucocorticoid therapy also appear to be at increased risk. Smoking is also a risk factor [6].

Most patients present with locoregional disease, and less than 20% of patients will present with or develop distant metastases. GIST with large tumor size and high mitotic activity are highly malignant, but the biological behavior of anorectal GIST is less clear [5, 15]. GIST of size <2 cm and mitosis <5 per 50 HPF were indolent, whereas those with size >5 cm and/or mitosis >5 per 50 HPF were highly malignant [5, 16]. Changchien identified age <50 and size >5 cm as independent prognostic markers [17].

GIST are best treated by surgery and are not radio- or chemosensitive. However, controversy exists whether abdominoperineal resection (APR) or conservative surgery is the best alternative [15]. Though the incidence of local recurrence is lower after APR, the distant metastasis and survival are not significantly different [17]. Patterns of recurrence and metastasis for anorectal GIST are the same as for GIST elsewhere, and the disease usually has a long or protracted course. Therefore long followups are essential and local recurrences if any can be reoperated, if resectable [15]. As regards adjuvant or salvage therapy imatinib mesylate, a tyrosine kinase inhibitor, has shown promising results in the management of patients with GIST [14]. Tumor responses to imatinib are seen in 80% of patients. However, kinase inhibition by imatinib is not uniformly successful [3]. It has been suggested that low risk GIST with size <2 cm and mitosis <5 per 50 HPF may be considered for local excision if sphincter saving surgery is technically feasible, and more aggressive GIST should be treated with radical excision [15].

Anorectal GIST, though rare, should be considered in the differential diagnosis of tumors in this region, especially if the pre-operative biopsy is equivocal. Gross and histopathological are both important, as prognosis depends on tumor size as well as grade. However, prognosis is usually better than for corresponding carcinomas in the region. Immunohistochemistry is a must, as CD-117 score is not only diagnostic but also guides adjuvant therapy and is an important prognostic marker.

Our case was a case of invasive anal canal GIST with tumor size more than 5 cm in maximum dimension. Mitotic figures were more than 5/50 HPF. The CD117—immunoreactive score was—3+ in spindled cells. Hence our patient has been put on adjuvant imatinib mesylate 400 mg daily.
